# Venocentric Lesions: An MRI Marker of MS?

**DOI:** 10.3389/fneur.2013.00098

**Published:** 2013-07-22

**Authors:** Matthew P. Quinn, Marcelo Kremenchutzky, Ravi S. Menon

**Affiliations:** ^1^Department of Medical Biophysics, Schulich School of Medicine and Dentistry, The University of Western Ontario, London, ON, Canada; ^2^Centre for Functional and Metabolic Mapping, Robarts Research Institute, The University of Western Ontario, London, ON, Canada; ^3^Department of Clinical Neurological Sciences, London Health Sciences Centre, The University of Western Ontario, London, ON, Canada

**Keywords:** venocentric lesions, multiple sclerosis, magnetic resonance imaging, T2^∗^ weighted imaging, susceptibility-weighted imaging

## Abstract

From the earliest descriptions of multiple sclerosis (MS), the venocentric characteristic of plaques was noted. Recently, numerous magnetic resonance imaging (MRI) studies have proposed this finding as a prospective biomarker for MS, which might aid in differentiating MS from other diseases with similar MRI findings. High-field MRI studies have shown that penetrating veins can be detected in most MS lesions using T2^∗^ weighted or susceptibility-weighted imaging. Future studies must address the feasibility of imaging such veins in a clinically practical context. The specificity of this biomarker has been studied only in a limited capacity. Results in microangiopathic lesions are conflicting, whereas asymptomatic white matter hyperintensities as well as lesions of neuromyelitis optica are less frequently venocentric compared to MS plaques. Prospective studies have shown that the presence of venocentric lesions at an early clinical presentation is highly predictive of future MS diagnosis. This is very promising, but work remains to be done to confirm or exclude lesions of common MS mimics, such as acute disseminate encephalomyelitis, as venocentric. A number of technical challenges must be addressed before the introduction of this technique as a complementary tool in current diagnostic procedures.

## Introduction

Demonstration of white matter lesions with magnetic resonance imaging (MRI) is central to multiple sclerosis (MS) diagnosis (1). However, white matter hyperintensities (WMHs) are not specific to MS and may reflect a number of physiological processes other than inflammatory demyelination (2). Requirements for MS diagnosis that demyelinating lesions be proved as disseminated in space and time should not overshadow the necessity for ruling out mimics of MS (3).

An early and accurate diagnosis with MS will allow quality of life to be retained through early initiation of *disease-appropriate* management (4). To that end, there is a growing interest in identifying biomarkers that can facilitate discrimination between MS and non-MS at first clinical presentation.

One such biomarker may be an MRI-detectable penetrating vein within a WMH. MS lesions have been known to be venocentric since the earliest descriptions of the disease (5). It was not until 2000 that Tan et al. directly demonstrated this physiological finding *in vivo* with MRI (6). Interest in this biomarker has grown in subsequent years. Many studies have explored the utility of MRI for discriminating between MS plaques and non-MS WMHs on the basis of central veins.

Are venocentric WMHs specific to MS? Can this information be used for earlier diagnosis at clinical field strengths? Claims that this biomarker “could overhaul current diagnostic algorithms” (7) in MS must be tempered until all data are vetted and the potential challenges with this technique are explored and overcome.

In this review, we first summarize relevant evidence in order to consider the specificity of venocentric lesions to MS. A relevant overview of imaging techniques is provided. Subsequently, our main objective is addressed: to review MRI studies which have explored imaging central veins in WHMs. Finally, we identify necessary challenges which must be overcome prior to bringing this biomarker into the clinic.

## Are Venocentric Lesions Unique to MS?

### Idiopathic inflammatory demyelinating disease

#### Multiple sclerosis

In 1868, Charcot reported that sclerotic plaques of MS were typically positioned along small veins, as determined from analyses of autopsy specimens (5). Dawson later described MS plaques that spread along prominent periventricular veins (8) (now known as Dawson’s fingers). Functionally, the vein is involved in the formation of the plaque as a necessary substrate for inflammation (9).

#### Acute disseminate encephalomyelitis

Acute disseminate encephalomyelitis (ADEM) is an immune-mediated demyelinating disease of the brain that has overlapping clinical and radiological features with MS (10). Indeed, a considerable portion of patients with suspected ADEM are eventually diagnosed with MS (11). Pathologically, the hallmark of ADEM is the narrow sleeve of perivenous inflammatory demyelination (12), differing from confluent demyelination in MS. It follows that lesions of ADEM, like those of MS, might appear perivenous on MRI – an uninvestigated issue.

#### Neuromyelitis optica

Like ADEM, neuromyelitis optica (NMO) is an immune-mediated demyelinating disease that is distinct from MS, but shares imaging and clinical features (13). A recent study has found that 35% of WMHs in NMO-spectrum disorders have an MRI-detectable penetrating vein, as opposed to 92% in MS (14).

### Neurological disease without primary demyelination

Thorough reviews are available which classify mimics of MS that may present with ambiguous clinical and radiological features (15), despite distinct underlying pathologies (vascular, autoimmune, metabolic, degenerative). There is limited literature describing venous involvement in pathological correlates of WMHs in these cases.

A limited number of studies have evaluated the presence of an MRI-detectable central vein in WMHs in the context of small vessel disease. Where one study reported the total fraction of venocentric lesions in Susac syndrome to be significantly reduced compared to MS (16), a separate study reported that WMHs in general microangiopathies and MS were equivalent in this regard (17). Given that Susac syndrome is ultimately a specific microangiopathy, questions are raised as to the consistencies of methodology of these studies.

Generally, rheumatic diseases such as systemic lupus erythematous, Sjögren’s syndrome, and Behçet’s syndrome have systemic presentations (18). When first presentation is neurological, WMHs are generally detected. For this reason, the neurologist must rule out these diseases when considering an MS diagnosis. Given modest overlap in components of disease pathology in neurological manifestations of rheumatic diseases and MS (autoimmune inflammation, vascular damage, foci of demyelination), it is imperative to address the unexplored possibility that WMHs in these systemic autoimmune diseases are venocentric.

## MRI of Veins

Owing to the small size of the veins that are found within MS lesions (on the order of 100s of microns), commonly used MRI venography techniques such as time-of-flight or phase contrast are insufficient for their detection.

### T2^∗^ weighted imaging

T2^∗^ weighted MR imaging forms the basis of detecting penetrating veins in MS lesions. T2^∗^ weighted images differ from T2 and T1 weighted in that they demonstrate increased sensitivity to inhomogeneous magnetic field (19). For a large range of magnetic fields within an imaging voxel, resonating spins will lose coherence rapidly, resulting in decreased signal intensity within that voxel.

Owing to the paramagnetic nature of deoxyhemoglobin (20), venous blood will perturb the local magnetic field. In voxels containing or adjacent to a vein, the spins will “see” a larger range of magnetic fields and, due to the above-described effect, cause reduced signal intensity for the voxel. Thus, in T2^∗^ weighted images, veins appear hypointense relative to surrounding tissues (21).

### Susceptibility-weighted imaging

Susceptibility-weighted imaging (SWI) is a technique that uses the phase data (typically discarded in clinical practice) to enhance the endogenous contrast in the T2^∗^ weighted magnitude image (22). In brief, the phase of MRI signal is proportional to the magnetic field and is thus also sensitive to the presence of venous blood. A phase “mask” can be created which, when multiplied with the magnitude image, improves the contrast of veins (i.e., makes veins more hypointense) without dramatically changing other contrast.

For both T2^∗^ weighted and SWI images, the visibility of a vein will depend not only on its size, but also on the dimensions of the imaging voxel. Best contrast is expected for a voxel that has a slice thickness that is two to four times the in-plane resolution (23). At 3 T, it has been estimated that parenchymal veins with diameters on the order of 100–200 μm can be detected for a 0.5 mm × 0.5 mm × 1.0 mm voxel (24).

### FLAIR/SWI fusion

The contrast of lesions may not be optimal on T2^∗^ or SWI images compared to FLAIR, the clinical standard for lesion detection. Two research groups have proposed multiplying the SWI phase mask with a co-registered FLAIR image to facilitate the simultaneous visualization of parenchymal veins and lesions (25, 26). Alternatively, one must identify lesions on FLAIR, and then toggle to a T2^∗^ weighted or SWI image to identify veins. It is uncertain if the increased post-processing required for FLAIR/SWI fusion will increase ability to detect penetrating veins in WMLs.

## Previous Work

A number of previous studies (Table 1) have undertaken the objective of visualizing veins in MS lesions, and can be generally considered in three categories: (1) those which seek to reproduce the observation that MS lesions tend to be venocentric, (2) those which seek to determine the specificity of this finding to MS, i.e., by determining if venocentric lesions are prevalent in other diseases, and (3) those which seek to determine the predictive value early in the disease course (for example, at the stage of clinically isolated syndromes) of venocentric lesions for conversion to MS. These are addressed below, in turn. As a metric, most studies have reported fractions of lesions with central veins to total lesions; herein reduced to percentage of lesions with central veins, or % LCV for clarity.

**Table 1:** **Venocentricity studies suggest MRI can be made sensitive to venocentric MS lesions. Specificity of this biomarker to MS is not well studied**.

Study	Field (T)	Subjects	Imaging	Voxel size	Metric	Outcome
Tan et al. (6)	1.5	17 CDMS	SWI post-gad	0.65 mm × 0.49 mm × 2.50 mm	% LCV	Periventricular WM: 100% deep WM: 98%
Ge et al. (27)	7	2 women with RRMS	T2* weighted	0.23 mm × 0.23 mm × 2 mm	%LCV	59%
Tallantyre et al. (28)	7	8 CDMS	T2* weighted	0.67 mm isotropic	% LCV	periventricular WM: 96% peripheral WM: 65%
Tallantyre et al. (29)	3, 7	7 CDMS, 7 HC	T2* weighted	7 T: 0.5 mm isotropic 3 T: 0.8 mm isotropic	% LCV	7 T: controls: 8%, MS: 87%; 3 T: MS only: 45%
Grabner et al. (25)	3, 7	10 patients with CDMS	FLAIR-SWI	0.3 mm × 0.3 mm × 1.2 mm	% LCV	25%
Lummel et al. (17)	3	15 MS, 15 microangiopathy	SWAN	0.52 mm × 0.52 mm × 2.6 mm	% LCV	MS: 80%; microangiopathy: 78%
Tallantyre et al. (30)	7	28 MS, 17 non-MS with white matter lesions (12 with vascular risk factors, 5 HC)	T2* weighted	0.5 mm isotropic	% LCV	MS: 80%, non-MS: 19%
Sinnecker et al. (14)	7	10 NMO-spectrum disorders, 18 MS	T2* weighted	0.5 mm × 0.5 mm × 2.0 mm	% LCV	NMO-SDs: 35%, MS: 92%
Wuerfel et al. (16)	7	5 Susac Syndrome (SS), 10 RRMS, 15 HC	T2* weighted	0.5 mm × 0.5 mm × 2.0 mm	% LCV	SS: 54%; MS: 92%, no lesions in HC
Kau et al. (31)	3	14 suspected MS; by follow-up, all were diagnosed (5 MS)	SWI	0.45 mm × 0.45 mm × 1.00 mm	CVS + lesions	All MS: ≥1 CVS + lesion; 8/9 non-MS, 0 CVS + lesions
Mistry et al. (7)	7	29 suspected MS; by follow-up, 22 were diagnosed (13 MS)	T2* weighted	0.5 mm isotropic	% LCV	All MS:>40% at baseline; all non-MS:<40% at baseline

*HC, healthy controls; % LCV, percentage of all lesions with central veins; CVS, central vein sign [the presence of a central vein in a large lesion (31)]*.

### Can central veins be detected in MS lesions?

The study of Tan et al. in 2000 was the first to use MRI to demonstrate that MS lesions are venocentric (6). Notably, this study is the only one thus far to attempt to do so at 1.5 T, and the results are remarkable. Using SWI acquired post contrast injection (to increase venous contrast in magnitude image), this study found % LCV to be 99. The authors emphasize that given the ubiquitous nature of small venules, only the correspondence of *ovoid* lesions specifically along a vein is typical of MS.

Subsequent studies in 2008 (27, 28) reproduced these findings at 7T using T2^∗^ weighted images, although the near-perfect correspondence between lesion and central vein was not reproduced. As in Tan’s study, Tallantyre et al. noted that nearly all lesions found near the ventricles were perivenous, an unsurprising finding given the high density of veins in this region. This potentially reduces the relevance of rating lesions in this area when considering an alternative diagnosis to MS, as lesions may be venocentric merely “by chance.”

Another study compared 7–3 T in terms of ability to demonstrate central veins in MS plaques using T2^∗^ weighted imaging, finding that only half as many lesions could be demonstrated as such using the lower field strength (29). This finding is puzzling considering the spectacular results from even lower field strength (1.5 T) noted above. Potentially, the large *isotropic* voxels used at 3 T in this study were not as sensitive to veins of diameters typical in MS lesions.

Grabner et al. used a fusion approach, multiplying a phase mask derived from 7 T imaging with a 3 T FLAIR image (25). Veins were found in only a quarter of lesions in the cohort of 10 MS patients. This result is in contrast to the otherwise consistent findings at 7 T using T2^∗^ imaging alone, suggesting FLAIR-SWI fusion may not yet be sufficiently refined for imaging this biomarker.

### Can central veins be detected in WMHs in other diseases?

Lummel et al. compared the % LCV between patients with MS and those with WMHs related to microangiopathy, finding there to be equally high prevalence of central veins within WMHs of the two groups (17). In stark contrast (as pointed out earlier), a later study compared MS patients against those with Susac syndrome, and found significant differences in % LCV between the two groups (16). In the latter study, the authors also investigated the presence of a hypointense lesion rim on T2^∗^, suggestive of iron deposition, which is known from histology. Whereas 41% of lesions in MS patients had a hypointense rim, this was detected in only 4% of patients with Susac syndrome.

A significant difference in % LCV between MS and NMO-spectrum disorders was noted in a different study (14). In this work, rim hypointensity was again reported, this time in roughly one quarter of MS patients, as opposed to 2% of NMO-spectrum disorder patients. As a biomarker, rim hypointensity appears to have low sensitivity but high specificity to MS.

With respect to asymptomatic WMHs, Tallantyre et al. reported less than a fifth had a central vein (with no subject above % LCV of 40%), whereas 80% of WMHs in MS did (with no subject below 40%) (30). The authors reported similar values of % LCV in all subtypes of MS. In this study, the non-MS group consisted of patients with known vascular risk factors as well as healthy controls. This group was substantially older than MS group [mean age (range), for non-MS: 60.8 (34–77); for MS: 46.5 (24–65)], making it impossible to separate out the effect of age as a risk factor for WMHs.

Given the above, the presence of a central vein has emerged as a highly sensitive marker of MS, with at least modest specificity in some cases. However, venocentricity of WMHs in numerous other diseases that often are considered against MS have yet to be studied in a similar way. This biomarker’s universal *specificity* to MS remains to be established.

### Is venocentricity predictive of an MS diagnosis?

Only two prospective studies to date have examined the predictive power of venocentric lesions for subsequent conversion to MS. In a 3 T SWI study, Kau et al. found that all study participants (but one) who, at baseline, had at least one lesion with a central vein later were diagnosed with MS, whereas all those with suspected MS at baseline without any venocentric lesions were later diagnosed with another disorder (31). However, the narrow inclusion criteria [at least 1 large (5 mm) lesion in a patient with suspected MS] obscure translation of this methodology to the clinic. Mistry et al. followed 29 patients with suspected MS (7). Diagnosis of MS by the follow-up at average of 26 months overlapped perfectly with % LCV>40% at baseline, as examined with 7 T T2^∗^ imaging. In both studies, the scope of non-MS diagnoses (i.e., at follow-up) was limited (mostly vascular disorders).

## Moving Forward

Recent evidence, especially from limited but exciting prospective studies, shows promise for the evaluation of venocentric lesions in aiding MS diagnosis. However, a number of challenges remain, described here.

### Clinical implementation

It has been established that 3 and 7 T MRI can demonstrate the presence of a central vein in many MS lesions. No study since the preliminary work of Tan et al. (6) (which lacked any control group or blinding) has investigated this promising biomarker at 1.5 T. While that study benefited from increased SWI contrast due to use of gadolinium, a trend in MS diagnostic criteria is to move away from dependence on such agents (1). It thus remains to be established whether central veins can be demonstrated in MS lesions at 1.5 T using conventional T2^∗^ imaging, or more likely, non-contrast SWI. Multi-echo modifications to SWI remain a possibility given their increased venous contrast (32), however such techniques come at the cost of increased scan time. Alternatively, given the increasing prevalence of 3 T MRI scanners in clinical use, optimization of a 3 T technique may be sufficient for centers where such a system is available.

### Imaging parameters

Venous visibility is intimately linked to voxel dimensions. Several studies reviewed here seemingly neglected the finding that isotropic voxels are not ideal for imaging small veins using SWI or T2^∗^ weighted contrasts (23) (see Table 1, “Voxel Size” column). As a note, a recent paper dealt specifically with the topic of T2^∗^ MRI at 3 and 7 T for small veins in MS plaques (33). Following numerical optimization and validation *in vivo*, the authors proposed revisions to echo times and voxel dimensions implemented in their previous studies to increase visibility of small veins within MS lesions (29, 30). Proposed voxel dimensions were incidentally in line with above-referenced previous optimizations.

### Venocentricity metric

The vast majority of studies reviewed here report some version of what we refer to as % LCV. Given the demonstration of a clean threshold in this metric to distinguish between MS and non-MS at baseline (7), this is an enticing approach. There are at least two challenges to face here.

The first challenge is: what to do in the case of patients with very small or large lesion burdens? In the first case, what is the minimum number of lesions that must be rated (and therefore be present) to obtain a confident evaluation? In the second case, might it be appropriate to rate only a subset of the lesions in order to obtain a representative but accurate % LCV? Using hypergeometric distribution methodology, Tallantyre et al. reported that if only 10 lesions per patient were rated (in patients with>10 lesions), the diagnosis of MS/non-MS could correctly be predicted with 90% certainty in 44 out of 45 patients tested (30).

The second challenge is: what constitutes a penetrating vein from a radiological standpoint? The shape of the lesion, and possibly the course of the vein within are likely relevant. In several studies, criteria for venocentricity are clearly and unambiguously outlined; in others, they are absent. It would be best to adopt a common set of criteria, such as those outlined in the works of the Nottingham group (28).

### Specificity of venocentric lesions to MS

As speculated above, it is possible that WMHs in non-MS diseases may contain MRI-detectable central veins. In this case, it may not be possible to discriminate against MS using the central vein biomarker reviewed here.

Ultimately, the number of diseases that are often differential diagnoses for MS is too large to allow systematic, disease-by-disease evaluation of typical % LCV, however common alternatives to MS should be vetted thoroughly. Given the known perivenous demyelination in ADEM (12), it is imperative that this disease be evaluated.

### Comparison with other disease markers

Para-clinical testing such as analyses of CSF, serum, visually evoked potentials, as well as demographic factors and specific symptoms also guide the diagnostic process in MS (1). It is yet to be determined if the venocentricity biomarker can offer additional diagnostic accuracy. Only then will its addition to the clinical toolbox be warranted.

## Conclusion

In the past decade, numerous studies have explored a promising biomarker for MS: MRI-detectable veins within lesions. This biomarker is well established as detectable at 3 and 7 T and efforts should be made to identify/optimize clinically practical methods for its evaluation. Prospective studies have shown that the presence of venocentric lesions at an early but ambiguous clinical presentation is highly predictive of future MS diagnosis. Work remains to be done to confirm or exclude lesions of common MS mimics as venocentric. Common imaging practice and lesion-rating paradigms should be adopted by scientists working in this field.

## Conflict of Interest Statement

Mr. Quinn and Dr. Menon report no conflicts of interest. Dr. Kremenchutzky or his institution received research support from the MS Society of Canada, Bayer, Biogen Idec, Genzyme, Serono, Teva Neuroscience, Sanofi, and Novartis.
